# Internet addiction and relationships with depression, anxiety, stress and academic performance among Egypt pharmacy students: a cross-sectional designed study

**DOI:** 10.1186/s12889-022-14140-6

**Published:** 2022-09-26

**Authors:** Moustafa Sayed, Christina Medhat Naiim, Marina Aboelsaad, Michael Kamal Ibrahim

**Affiliations:** 1 Clinical Pharmacy Practice Department, Faculty of Pharmacy, The British University in Egypt (BUE), El-Sherouk City, P.O. Box 43, Cairo, Egypt; 2 Center of Drug Research and Development (CDRD), Faculty of Pharmacy, The British University in Egypt (BUE), El-Sherouk City, Cairo, Egypt; 3 Department of Developmental Pharmacology, Egyptian Drug Authority (EDA), Giza, Egypt

**Keywords:** Internet addiction, Depression, Anxiety, Academic performance

## Abstract

**Background and Aims:**

Pharmacy students represent the future of healthcare professionals and with daily use of the internet for different activities has made internet addiction (IA) of a growing concern. The main objectives of this study were to 1) assess internet addiction among pharmacy undergraduate students as well as factors associated with it; 2) assess the relationships between internet addiction and common mental disorders (depression, anxiety, and stress), in addition to academic performance and body mass index factors.

**Methods:**

We utilized a cross-sectional questionnaire that was conducted among 808 students of Egypt university pharmacy students across the country. The surveys used included: Young Internet Addiction Test (YIAT) and the Depression Anxiety Stress Scales (DASS 21).

**Results:**

YIAT average score was 44.75 (19.72%); prevalence rate of potential IA was 311 (38.5%) with no gender significant difference. We couldn’t detect any type of correlation between potential IA and GPA. However, a robust correlation was found between internet addiction vs depression, anxiety and stress collectively.

**Conclusions:**

Internet addiction is usually associated with mental related disorders thus it is of paramount important to identify it among students. Different therapeutic interventions could include management to IA and common psychological disorders such as depression, anxiety, and stress.

## Introduction

Internet usage has grown to more than 2.5 billion users worldwide [1] affecting mainly the young generation [2]. During the last decade, internet has become the essential tool for people to find information and communicate with each other. Yet, some people became addicted to the internet especially the young adults [3]. Internet addiction is defined as the excessive use of the internet in an uncontrolled time consuming manner which leads to timelessness and disruption of people's lives [4]. Such behavior may lead to psychological, social or personal adversities [5].

Some diagnostic criteria for internet addiction was developed by Yong et al. [6] to assess the core symptoms associated with internet addiction such as tolerance, impairment of control, preoccupation and excessive online time. The prevalence may vary according to age, sex and ethnicity, and is more common among college students [7]. A high prevalence of personality problems is observed in internet-addicted people [8, 9]. Mood disorders, poor sleep quality, impulsivity, low self -esteem, suicide, decreased physical activity, and health problems (migraine, back pain, obesity) are also reported to be associated with extensive use of internet [10–13].

According to World Health Organization (WHO), psychological disorders are one of the disability causes in the world [14]. Mental health problems include depression, anxiety and stress. Each one of them is considered a threat to the public health especially in the young population. Stress is considered a threat to the well- being of humans. It occurs whenever life's burdens exceed the person ability to withstand them resulting in mental and physiological changes that my alter their health and causes many diseases [15]. Anxiety, on the other hand, is seen as both a psychological and a physiological state as it affects the brain, physical activity, emotions, and behavior. Uneasiness, fear or worry all together are the consequences of stress [16]. If these symptoms persist, the person is at risk of developing depression. Depression is the feeling of guilt, worthlessness, irritability, restlessness and loss of interest in life pleasures. [17]

University students undergo a transitory period from being teenagers to become adults. Such transition can cause a lot of stress, depression and anxiety to some of them. A study conducted by Kavithah et al. and Amr et al. [18, 19] predicted the prevalence rates of depression and anxiety among students in Al-Mansura University, 28.3% and 21.2% respectively. Likewise, data were presented by Ibrahim et al. [20] suggested that 37.6% of Asyut University students suffered from moderate or severe depressive symptoms. Furthermore, a study was carried out in Saudi Arabia predicted the depression, anxiety and stress prevalence rates among its university students 48.1%, 58.9%, and 40.4% respectively [21]. Previous research studies assessed the correlation between emotional status and different factors may affect students such as weight, academic performance, internet addiction, and gender difference.

Many research studies addressed academic life stress among undergraduates as one of the causes of mental disorders. [22–29] It is crucial to determine the risk factor and prevalence of stress that medical students encounter almost every day in which affects their mental health, academic performance, and consequently their physical health. Abdulghani et al. [22] reported high stress rates associated with physical problems among medical students especially female students. Similarly, another study was carried out by [30] confirmed that stress and anxiety prevalence rates are higher in medical students in comparison with the nonmedical ones.

Previous research studies discussed the relation between internet addiction and different mental disorders. A study was conducted in Lebanon revealed a significant correlation between internet addiction and DASS scores, where IAS was 16.8% among college students [31]. Another study was conducted in Turkey also revealed that 27% of undergraduate medical students were addicted to internet [32].

Among the major public health concerns that require a thorough attention are overweight and obesity. Unnecessary fats deposited in the body lead to such conditions. Thus, calculating the measure of obesity is crucial to prevent any health problems. For example, obesity is defined as using the body mass index (BMI) equation, where a person’s weight (in kilograms) or (in pounds) and his or her height (in meters) or (in inches) plugged in an equation to determine whether he or she is obese or overweight. Usually a person with a body mass index equals to 30 is obese, while the person with a BMI equals to or more than 25 is overweight. [33]

According to the World Health Organization (WHO), obesity has now become the highest prevalence in all the World Health Organization regions not just in the Americas. Statistics reveal that 62% of adults are overweight or obese. This epidemic did not only affect adults but also children and adolescents, in which 20% to 25% are either overweight or obese. Lung disease, diabetes mellitus, type-2 diabetes, sleep difficulty, cardiovascular diseases, musculoskeletal disorders, and low self-esteem are all consequences to overweight and obesity. In addition, overweight children have a higher risk of staying overweight or obese as adults. [34]

The growth of obesity is also remarkable according to Centers for Disease Control and Prevention (CDC), 39.8% of adults above 20 has obesity (2015–2016), and 71.6% of adults aged above 20 suffer from overweight (2015–2016) (“Obesity and Overweight,” n.d.) in US. Previous studies have provided a comprehensive analysis of associations between obesity and different emotional status. Based on the global statistics on mental health, around 1-in-7 people in the world, which falls within (11–18%), suffer from one or more mental disorders. Worldwide, around one billion people in 2017 have experienced one. The prevalence in Egypt is 14.57% while in United States is 17.34% (2017). [34]

Eliacik et al. have claimed a relation between internet addiction and degree of obesity during adolescence [35]. It is well known that obese individuals are more prone to be depressed, have lower performance in school compared to healthy peers. [36]. Insomnia and short sleep duration is associated with high food intake and internet use [37, 38]. Sedentarism has been associated with excessive use of internet which is related to decreased energy expenditure or lower physical activity [39–41].

A study was conducted by (Peltzer et al., 2014) [42] to assess the prevalence of overweight and obese people among students enrolled in different universities in the world. The data suggested that Egyptian university students have higher rates of obesity especially females. Similarly, another study conducted in Saudi Arabia revealed high rates of overweight and obesity especially in the female group. [43] In addition, others have reported higher rates of obesity among male university students in Saudi Arabia. [44, 45]

Research has lately focused on the link between emotional status and obesity. Most of the researches done before that focused mainly on obesity and depression. However, other psychiatric morbidities, other than depression, should be taken into account. In Arab countries, recent studies from Egypt, Saudi Arabia, and United Arab Emirates reported high rates of anxiety and depression. The mental disorders in Egypt are associated with many factors such as demographic, social, behavioral and educational factors. Much of the literature suggested that Egyptian medical students are at higher risk of developing emotional status problems than any other groups. Academic stress is one of the risk factors they encounter every day. [17]

Our hypothesis was that internet addition is a serious problem among pharmacy students, examining its association with the emotional status is of great concern to take the proper measures to address this issue. Internet addiction may affect the emotional and mental status of students through depression, anxiety and stress and thus affect their overall academic performance and their career goals.

The objectives of this study are: 1) Assess potential internet addiction using (Kimberly S.Young, 1995) in pharmacy students among different universities in Egypt, as well as socio-demographic factors and other factors associated with it; 2) Assess the relationship between potential internet addiction and different common mental disorders such depression, anxiety, and stress among students using DASS as a valid questionnaire.

### Objectives

The objectives of this study are to determine prevalence and correlation of emotional status, body image and internet addiction with academic performance among Egyptian pharmacy students.

## Methods

### Study design and participants

The cross-sectional study conducted on pharmacy students in all grade levels during the academic year 2019–2020 (November 2019-May 2020). The mean age of students participated was 21.16 (1.64%).

### Sample size

The study targeted pharmacy students in Egypt. The total number of students participated in the study is 808.

### Study instruments

Students were asked to fill out on campus and online self-reported questionnaires which took approximately 10 min to answer and collected data on: demographics, anthropometrics, psychological assessment and internet addiction.

### Demographic data

Age, gender, university, college, academic year, and grade /GPA performance.

### Anthropometric measurements

The body weight in kilograms and height in meters reported by each participant. According to WHO, the body mass index was calculated as body weight in kg divided by height in m.^2^. BMI of 18.5 is classified as underweight and 25–29.9 is overw eight and BMI of 30 is considered as obese. [46]

### Psychological assessment

Pharmacy students were subjected to the Arabic and English version of Depression Anxiety Stress Scales (DASS-21). It is composed of three-self report scales designed to measure the different emotional status. The 21 DASS questions were adjusted according to their importance and the Egyptian students’ culture. Each of the three DASS contains seven items, scored on a four- point scale to rate the extent to which participants have experiences each state over the past few weeks. Corresponding answers for each items are scored 0–3 as follow: 0 indicates " Did not apply to me at all" – 1 indicates "Applied to me to some degree, or some of the time" – 2 indicates " Applied to me to a considerable degree or a good part of time" – 3 indicates "Applied to me very much or most of the time".

### Internet addiction diagnostic questionnaire

To test the extent of internet addiction among pharmacy students, the internet addiction diagnostic questionnaire (Kimberly S.Young, 1995) was used. The questionnaire consists of 20 questions that evaluate the symptoms on a five-point scale (0 = Does not apply, 1 = seldom, 2 = sometimes, 3 = often, 4 = often, 5 = always). The numbers for each response were added to determine the final score after all questions were answered. The higher the score, the greater the degree of addiction and concerns emerging from internet use.

We performed Cronbach’s alpha test validate the reliability of the questionnaires used. Alpha values for all questionnaires used were between 0.8–0.89, which means the internal consistency is good for all items in the questionnaires. **(**Table 1).

**Table 1:** Cronbach’s-Alpha values for the DASS-21 subscales and IAT

Scale	Number of items	Cronbach’s-Alpha
Depression	7	0.85
Anxiety	7	0.8
Stress	7	0.83
IAT	20	0.89

Cronbach’s-Alpha values for all scales indicates the high internal consistency and validity of the questionnaires

### Statistical analysis

The statistical analysis was performed using SPSS software for Windows (version 20.0). At 0.05, the significance level was set. The mean and the standard deviation (SD) for continuous variables and percentage for categorical variables were used to summarize sample characteristics. Internet addiction users were categorized as normal or potential internet users. Multivariate analysis was necessary to determine the effect of multiple explicative explanatory variables and which of the explanatory factors act independently on the internet addiction.

Univariate analysis independent variables were conducted using the Chi-square independence tests or Fisher Exact test. Subsequently, logistic regression analysis was done with the dichotomized internet addiction as the dependent variable. According to the Enter method participants’ characteristics and scores that showed associations with *p*-value < 0.25 in univariate analysis, were candidates for the multivariate model. Collinearity among independent variables was verified, expressed as variance inflation factor.

### Ethical considerations

Before study start-up, permission was taken from the ethics committee of the Faculty of Pharmacy at the British University in Egypt. The participants were briefed about the study rationale and were reassured about the confidentiality safeguards for their personal information and responses. The answered survey submission was considered as consent for study participation. Respondents’ participation was completely consensual, anonymous and voluntary.

All methods were performed in accordance with the relevant guidelines and regulations by the Research Ethics Committee, Faculty of Pharmacy, the British University in Egypt, Cairo, Egypt (protocol # EX-1502). Informed consent was obtained from all participants who filled out the online questionnaires during this study.

## Results

### Subjects’ characteristics

During the course of this study, a total of 808 participants completed the devised online survey questions. Among these, the average age (measured in years) was 21.6 ± 1 and 594 students (73.5%) were females. When identifying the academic performance, only 15 students (1.9%) got a GPA of A (3.6 out of 4 and higher) while 657 (81.3%) got a GPA of B and B + (Table 2). With regards to the students’ BMI, results disclosed that 509 students have normal BMI (63%) while 274 students have a BMI of overweight and higher (33.9%).

**Table 2:** Demographic characteristics of sample population (*N* = 808)

Variable	Number (%)
**Gender**	
Male	214 (26.5)
Female	594 (73.5)
**Age**	
18–19	132 (16.3)
19–20	183 (22.6)
20–21	143 (17.7)
21–22	171 (21.2)
22–24	179 (22.2)
Mean age (SD)	21.16 (1.64)
**GPA**	
A	15 (1.9)
B^+^	411 (50.9)
B	246 (30.4)
C^+^	76 (9.4)
C	52 (6.4)
D	8 (1.0)
**BMI**	
Thin	25 (3.1)
Normal	509 (63)
Overweight	212 (26.2)
Obesity I	51 (6.3)
Obesity II	7 (0.9)
Obesity III	4 (0.5)

### Emotional status and internet addiction among Egypt pharmacy students

The mean score of DASS depression scale was 8.85 (5.26%). In particular, 20.7% of the students reported an extremely severe depression status, while 23.6% displayed a normal status (Table 3). With respect to the level of anxiety, 20.5% of the students reported an extremely level of anxiety, 12.1% reported a severe anxiety level, 15.2% a moderate level while 34.9% reported a normal level of anxiety (Table 3). Regarding stress level among students, 8% of the student reported an extremely severe level of stress, 14.1% severe level of stress, 19.7% reported a moderate level of stress and 44.1% reported to have a normal level (Table 3). On the other hand, IAT showed that 38.5% of the students have a potential for internet addiction which represents a high risk factor that may affect emotional status, social activities and academic performance among pharmacy students in Egypt (Table 3).

**Table 3:** Number and percentage of students in each category of the applied questioners: DASS and IAT (*N* = 808)

Item	N (%)	Cut-off Point
**DASS D**		
Normal	191 (23.6)	0–4
Mild	113 (14)	5–6
Moderate	210 (26)	7–10
Severe	127 (15.7)	11–13
Extremely severe	167 (20.7)	14 +
Mean score (SD)	8.85 (5.26)	
**DASS A**		
Normal	282 (34.9)	0–3
Mild	139 (17.2)	4–5
Moderate	123 (15.2)	6–7
Severe	98 (12.1)	8–9
Extremely severe	166 (20.5)	10 +
Mean score (SD)	5.97 (4.5)	
**DASS S**		
Normal	356 (44.1)	0–7
Mild	114 (14.1)	8–9
Moderate	159 (19.7)	10–12
Severe	114 (14.1)	13–16
Extremely severe	65 (8.044)	17 +
Mean score (SD)	8.72 (4.92)	
**IAT**		
Normal internet use	497 (61.5)	0–30
Potential internet addiction	311 (38.5)	50–100
Mean score (SD)	44.75 (19.72)	

*D* Depression, *A* Anxiety, *S* Stress, *IAT* Internet Addiction Test

### Univariate analysis

The univariate analysis showed that potential internet addiction was not significantly different between males and females (*p*-value = 0.297). GPA, depression, anxiety and stress were significantly related to potential internet addiction (*p*-value = 0.03, < 0.001, < 0.001, < 0.001 respectively); however, neither age nor BMI, was significantly related to internet use (Table 4).

**Table 4:** Univariate analysis of the relationships between potential internet addiction and participants’ characteristics (*N* = 808)

		**Normal internet use (*N* = 497)**	**Potential internet addiction *N* = 311)**	***p*-value**
**Gender**				0.297 ^$^
	Male	138 (64.5%)	76 (35.5%)	
	Female	359 (60.4%)	235 (39.6%)	
**Age**				0.135 ^$^
	18	77 (15.5%)	55 (17.7%)	
	19	106 (21.3%)	77 (24.8%)	
	20	86 (17.3%)	57 (18.3%)	
	21	103 (20.7%)	68 (21.9%)	
	22–24	125 (25.2%)	54 (17.3%)	
**GPA**				**0.03 **^**#**^
	A	9 (1.8%)	6 (1.9%)	
	B^+^	265 (53.3%)	146 (47%)	
	B	156 (31.4%)	90 (29%)	
	C^+^	34 (6.9%)	42 (13.5%)	
	C	28 (5.6%)	24 (7.7%)	
	D	5 1%)	3 (0.9%)	
**BMI**				0.592 ^#^
	Thin	13 (2.6%)	12 (3.9%)	
	Normal	318 (64%)	191 (61.4%)	
	Over wt	128 (25.8%)	84 (27%)	
	Obesity I	30 (6%)	21 (6.7%)	
	Obesity II	4 (0.8%)	3 (1%)	
	Obesity III	4(0.8%)	0 (0%)	
**Depression**				** < 0.001 **^**$**^
	Normal	158 (31.8%)	33 (10.6%)	
	Mild	76 (15.3%)	37 (11.9%)	
	Moderate	133 (26.7%)	77 (24.8%)	
	Severe	66 (13.3%)	61 (19.6%)	
	Extremely severe	64 (12.9%)	103 (33.1%)	
**Anxiety**				** < 0.001 **^**$**^
	Normal	214 (43%)	68 (21.8%)	
	Mild	90 (18.1%)	49 (15.8%)	
	Moderate	81 (16.3%)	42 (13.5%)	
	Severe	49 (9.9%)	49 15.8%)	
	Extremely severe	63 (12.7%)	103 (33.1%)	
**Stress**				** < 0.001 **^**$**^
	Normal	274 (55.1%)	82 (26.3%)	
	Mild	73 14.7%)	41 (13.2%)	
	Moderate	78 (15.8%)	81 (26%)	
	Severe	50 (10%)	64 (20.7%)	
	Extremely severe	22 (4.4%)	43 (13.8%)	

^$^Chi square test of association

^#^Fisher’s exact test

*GPA* is the average of all the grades students receive during their undergraduate study with a *GPA* of 4.0 meaning that the student has earned all As in his or her classes.

### IA and depression, anxiety and stress

IA was clearly associated with all the three variables of DASS 21 score. In bivariate analysis, there was a strong positive correlation between IAT scores and depression, anxiety and stress scores. Moreover, depression, anxiety and stress scores also correlated positively with each other (Table 5). On the other hand, a weak negative correlation was observed between IAT and GPA.

**Table 5:** Correlation analysis between population characteristics and IAT score

		Gender		Age		GPA		BMI		DASS D		DASS A		DASS S		IAT score
	Gender		1.000		-.063-		.059		-.089-^*^		.084^*^		.089^*^		.037	.041
					.072		.092		.012		.017		.011		.298	.244
			808		808		808		808		808		808		808	808
	Age		-.063-		1.000		.152^**^		.007		.005		-.023-		.006	-.040-
			.072				.000		.836		.881		.514		.862	.256
			808		808		808		808		808		808		808	808
	GPA		.059		.152^**^		1.000		.026		-.048-		-.041-		-.030-	-.091-^**^
			.092		.000				.453		.177		.249		.397	.009
			808		808		808		808		808		808		808	808
	BMI		-.089-^*^		.007		.026		1.000		.044		-.005-		.058	-.006-
			.012		.836		.453				.209		.898		.102	.874
			808		808		808		808		808		808		808	808
	DASS D		.084^*^		.005		-.048-		.044		1.000		.608^**^		.684^**^	.362^**^
			.017		.881		.177		.209				.000		.000	.000
			808		808		808		808		808		808		808	808
	DASS A		.089^*^		-.023-		-.041-		-.005-		.608^**^		1.000		.642^**^	.350^**^
			.011		.514		.249		.898		.000				.000	.000
			808		808		808		808		808		808		808	808
	DASS S		.037		.006		-.030-		.058		.684^**^		.642^**^		1.000	.358^**^
			.298		.862		.397		.102		.000		.000			.000
			808		808		808		808		808		808		808	808
	IAT Score		.041		-.040-		-.091-^**^		-.006-		.362^**^		.350^**^		.358^**^	1.000
			.244		.256		.009		.874		.000		.000		.000	
			808		808		808		808		808		808		808	808

^**^Correlation is significant at the 0.01 level (2-tailed)

^*^Correlation is significant at the 0.05 level (2-tailed)

### Logistic regression model

The logistic regression model showed that age, depression and stress were significantly associated with internet addiction. In other words, it was found that the psychological variables of depression and stress, but not anxiety, were significant predictors of IA. Also, GPA score was observed as a predictor for IA (Table 6).

**Table 6:** Multiple logistic regression for the predictors of internet addiction in pharmacy students

Predictor	B	df	*p*	OR	95% CI for OR		Co-linearity statistics	
					**Lower**	**Upper**	**Tolerance**	**VIF**
Age	-.129-	1	.008	.879	.799	.966	.982	1.018
GPA	-.250-	1	.101	.779	.577	1.050	.974	1.027
Depression	.057	1	.011	1.059	1.013	1.107	.404	2.477
Anxiety	.043	1	.078	1.044	.995	1.095	.475	2.104
Stress	.082	1	.002	1.086	1.030	1.144	.335	2.984
Constant	1.550	1	.161	4.711				

## Discussion

The goal of this study was to find out how common potential IA is among Egyptian university pharmacy students, to assess correlations between IA and participant characteristics (primarily age, gender, GPA, and BMI), and to look into probable links between IA, anxiety, depression, and stress. Our research found that IA was unrelated to gender, age, or GPA, and that 38.5 percent of individuals had IA symptoms, with a mean YIAT score of 44.75. These findings are similar to those previously published for young adults. [47, 48] In addition, different research had been performed in Asia showed such significance. [49, 50] Some research mentioned that IA incidence turned into better in males [51], while others did not find difference between genders [52]. Some studies have shown that internet addiction could affect students' studying performance as students tend to spend much of their time using the internet.

[53–56]. However, that was not the case in our finding as we found a weak negative correlation between IA and GPA, which contradicts others’ findings. This could be explained that the time of internet use reported by the students in this study could have a big part of it invested in their studies, meaning that they spend more time on the internet to get their projects or assignments done.

One of the objectives of the present research was to study the impact of gender on the scores of internet addiction behavior. Schere et al. reported that male gender is a significant predictor of the internet addiction. However, we have reported that gender is not correlated to IA. This could be explained that maybe decades ago there was a difference between male and female use of internet but looks like there is no difference these days.

When analyzing despair, our effects additionally confirmed that 20.7% of individuals suffered from clinically giant stage of despair and a sturdy correlation became discovered among ability net dependency and despair. Depression occurrence stated on this examine is regular with the character of the pattern studied (younger students) and is similar to what's stated in teens elderly 18 to 23 [57]. Anxiety is commonly taken into consideration terrible results or headaches of net addiction [17].

Detecting elements related to educational overall performance in college students is of huge significance due to the fact an inverse relation exists among educational overall performance, stress, depression, tension and internet addiction [58].

### Strength and limitations

Our findings must be viewed in light of the study's methodology and limitations. We based our findings on self-reported behaviors. The most often used instruments for measuring physical and mental health evaluation subjective problems are self-reporting questionnaires. [59–61] The self-report method represents the interviewee's point of view and may be more appropriate for reporting.

The questionnaires were designed in a scale pattern to encourage answer and with a shorter interview period to prevent disrupting the students, in the hopes that the questionnaire's simplicity would make it easier for respondents to provide correct data. Notwithstanding these limitations, the findings observed in this study are important and warrant further investigations.

To the best of our knowledge, this is the first study to examine the relationship between three psychosocial stressors in Egyptian university pharmacy students: anxiety, depression, stress, and IA. Because this addiction frequently coexists with other psychological issues, and IA could be one visible tip of a complicated iceberg, our findings highlight the need of identifying and providing assistance to students with possible IA.

## Conclusion

Internet addiction is usually associated with mental related disorders thus it is of paramount important to identify it among students. Different therapeutic interventions could include management to IA and common psychological disorders such as depression, anxiety, and stress. Based on that it is advised to increase awareness among students on the internet associated health risks, which could affect their mental health status. Moreover, universities and colleges should offer psychiatrist office services to provide continuing counseling for students. Further studies in this field should throw light on relation between IA and drug abuse and other illicit drug consumption among college students.

## Data Availability

The datasets generated and/or analyzed during the current study are not publicly available due to patients’ privacy but are available from the corresponding author on reasonable request.
